# Effectiveness of a quality management program in dental care practices

**DOI:** 10.1186/1472-6831-14-41

**Published:** 2014-04-28

**Authors:** Katja Goetz, Stephen M Campbell, Björn Broge, Marc Brodowski, Michel Wensing, Joachim Szecsenyi

**Affiliations:** 1 Department of General Practice and Health Services Research, University of Heidelberg, Vosstr. 2, Building 37, Heidelberg D-69115, Germany; 2 Centre for Primary Care, Institute of Population Health, University of Manchester, Manchester, UK; 3 AQUA-Institute for Applied Quality Improvement and Research in Health Care, Goettingen, Germany; 4 Scientific Institute for Quality of Health Care, Radboud University Nijmegen Medical Centre, Nijmegen, The Netherlands

**Keywords:** Dental care, Oral health care, Quality of care, Quality improvement, Quality management

## Abstract

**Background:**

Structured quality management is an important aspect for improving patient dental care outcomes, but reliable evidence to validate effects is lacking. We aimed to examine the effectiveness of a quality management program in primary dental care settings in Germany.

**Methods:**

This was an exploratory study with a before-after-design. 45 dental care practices that had completed the *European Practice Assessment* (EPA) accreditation scheme twice (intervention group) were selected for the study. The mean interval between the before and after assessment was 36 months. The comparison group comprised of 56 dental practices that had undergone their first assessment simultaneously with follow-up assessment in the intervention group. Aggregated scores for five EPA domains: ‘infrastructure’, ‘information’, ‘finance’, ‘quality and safety’ and ‘people’ were calculated.

**Results:**

In the intervention group, small non-significant improvements were found in the EPA domains. At follow-up, the intervention group had higher scores on EPA domains as compared with the comparison group (range of differences was 4.2 to 10.8 across domains). These differences were all significant in regression analyses, which controlled for relevant dental practice characteristics.

**Conclusions:**

Dental care practices that implemented a quality management program had better organizational quality in contrast to a comparison group. This may reflect both improvements in the intervention group and a selection effect of dental practices volunteering for the first round of EPA practice assessment.

## Background

Improving the quality of healthcare is a high priority in Western health care systems [1] driven by factors such as reducing adverse events, optimizing efficiency, and enhancing patient satisfaction [2]. An excellent definition of quality in healthcare is given by Mills & Batchelor [3]. In essence, however, quality of care can been defined and evaluated in terms of structure, process and outcomes [4]. Whilst the presence of specific organizational structures does not necessarily result in better clinical processes and outcomes, organizational aspects are certainly enablers of higher performance [4,5]. Little is known about how to improve quality of organizational aspects of primary sector dental care. The majority of literature and the evidence base for defining and measuring quality in primary care come from general medical practice and not from oral health care settings [6]. However, assessing and monitoring the quality of dental care play an important role in quality assurance and quality improvement [7].

In most health care systems, a variety of quality improvement initiatives have been implemented to enhance both health care management broadly speaking and dental health care specifically speaking [3,7-10]. For instance, in the United Kingdom quality indicators were developed for the new National Health Service (NHS) dental contract which targets measuring the quality of patient care as well as performance [3]. In 2005, in Scotland, the “Action Plan for Improving Oral Health and Modernising NHS Dental Services” was announced [11]. Since 1997, in the United States, an assessment instrument developed and initiated by MetLife has been implemented for dental care providers [12]. Particularly countries such as the United Kingdom, the United States and Canada have shown expertise in development and implementation of quality management systems. Quality management means quality assurance: the systematic measurement and monitoring of process, structure and outcome of care and results in a continuous improvement process. For example, the plan-do-study-act cycle, to ensure quality of care [9,13]. In 2006, the German government stipulated that general dental practitioners should implement a system of annual assessment of quality management, in the same way that general medical practices are expected to do [14]. Although, to date, there are no formal sanctions, so participation remains voluntary. The result has been that different quality management systems have become available for health care providers in primary care settings [15]. These different quality management systems measure structure and process of care as well as non-clinical outcomes of patients.

However, while such quality management programs are available for dental care, evidence on their impact and effectiveness is sparse [6], with some exceptions [15,16]. There is an urgent need for validated quality assessment tools for dental care [7]. The *European Practice Assessment* is a comprehensive, integrative and multifaceted tool for quality assessment and quality improvement in health care in terms of quality management. It is based on quality indicators developed for use in primary medical care settings to evaluate the structure and process of care [17]. The *European Practice Assessment* tool has shown effectiveness in improving the management of general medical practices [18,19]. The current study focuses on the implementation and repeated measurement of *European Practice Assessment* tool in primary dental care settings and examined whether improvements occurred in dental care practice that completed the *European Practice Assessment* twice compared with dental care practices that completed the *European Practice Assessment* once.

## Methods

### European practice assessment

In 2004, the *European Practice Assessment* was developed for general medical care and already in 2005, the content as well the process was adapted for general dental care settings [17,19,20]. An expert group consisting of dentists and employees of the Institute for Applied Quality Improvement and Research in Health Care (AQUA-Institute) adapted and piloted the *European Practice Assessment* for dental care practices. In both cases, the *European Practice Assessment* consists of a set of validated quality indicators for external and internal assessment including a patient survey of satisfaction with care and a staff job satisfaction survey, an outreach visit by a trained external facilitator, structured feedback, a team-meeting in the practice and formal accreditation by an external organization [17]. These different indicators are framed within five key conceptual domains: “infrastructure”, “information”, “finance”, “quality and safety” and “people”. Some indicators for dental care practices are different to those for general medical care. The domain “infrastructure” was expanded by dimensions ‘material management’ and ‘laboratory management’; the domain “quality and safety” by dimensions ‘safety of staff and patients, hygiene, infection control’ and ‘provisions for emergency situations’; and the domain “information” by dimensions ‘communication with other health care providers’ and ‘information for patients on practice, practice policy and community resources’. Detailed information about the European Practice Assessment for dental care settings on a website is under construction.

After the implementation of *European Practice Assessment* in either a general medical care practice or a dental care practice, feedback is given by the trained facilitator using software called *Visotool*, which shows the results of the assessment. An anonymous comparison between a practice’s score and all other practices that have undertaken the assessment is available, which serves as a catalyst for quality improvement and for benchmarking. The application of the *European Practice Assessment* is coordinated by the AQUA-Institute based in Goettingen, Germany [21].

### Design and participants

**Figure 1:**
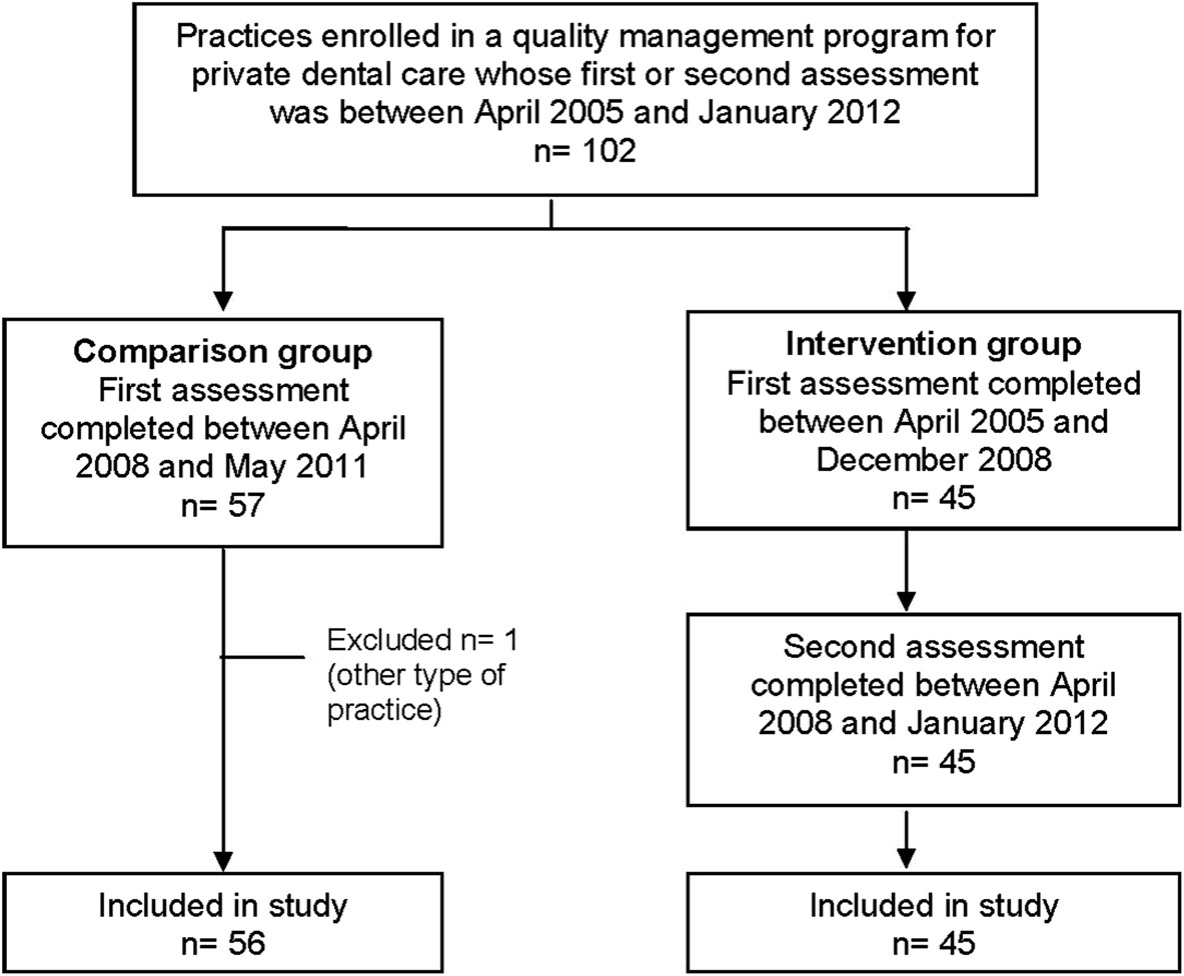
Selection of private dental care practice for the intervention and comparison.

The study conforms to the STROBE-Guidelines. A before-after study design was used with an intervention group and a comparison group of dental care practices. We included dental care practices in Germany that had completed the *European Practice Assessment* as part of a quality management program. For the intervention group, we identified all 45 dental care practices which had completed the *European Practice Assessment* twice, with their first assessment between April 2005 and December 2008 and their second assessment between April 2008 and January 2012. The interval between first and second assessment was around 36 months. During the period between April 2008 and May 2011 when the intervention practices were undergoing their second assessment, 57 dental care practices were undergoing their first assessment with the *European Practice Assessment* program but none had yet had a second assessment. These were called upon as our comparison group. One dental care practice was integrated into a general medical care centre and was excluded from the analysis. Therefore, 56 practices in total were included in the comparison group. Figure 1 shows the distribution of dental care practices to intervention and comparison groups respectively.

### Statistical analysis

The analyses were performed using SPSS version 20.0 (SPSS Inc., IBM). Continuous data were summarized using means and standard deviations. Categorical data were presented as frequency counts and percentages. The practice characteristics of the intervention and comparison groups were compared using Students t-test for continuous data and Chi^2^ test for categorical data.

Z scores were used to compare the overall mean score across all domains and dimensions of the *European Practice Assessment* instrument, in the intervention (second assessment) and comparison groups. Z scores were used to normalize raw scores [18,22]. The mean scores of all domains and dimensions were based on the proportion of indicators for which a positive response was achieved by all of the practices, on a scale of 0 to 100. The 95% confidence intervals were calculated for the differences in scores between the first and second assessment and for the differences between the second assessment and the comparison group. Furthermore, linear regression analyses were performed with aggregated scores on each of the five domains as dependent outcomes; practice characteristics as well as the affiliation to intervention (second assessment) or comparison group were handled as potential predictors. An alpha level of p < 0.05 was used for tests of statistical significance.

### Ethics approval

Ethical approval was not required because we used secondary data available from the routine implementation of a quality management program in primary dental care sector in Germany [14]. All elements of the *European Practice Assessment* and the information from the trained external facilitators were anonymized for data analysis in our study. No additional information or data from patients or staff were collected.

## Results

The practices included in the intervention (second assessment) and comparison groups did not differ significantly in the relevant covariates of practice characteristics (Table 1).

**Table 1:** Characteristics of the study sample

**Characteristics**		**Intervention**	**Comparison**	p-value*
		**group (n = 45)**	**group (n = 56)**	
Mode of practice; n (%)	single	32 (71.1)	38 (67.9)	0.10
	group	13 (28.9)	17 (30.3)	
	missing	--	1 (1.8)	
Location of practice; n (%)	urban	19 (42.2)	22 (39.3)	0.19
	rural	24 (53.3)	30 (53.6)	
	missing	2 (4.4)	4 (7.1)	
No. of dentists	mean	1.3	1.4	0.71
	SD	0.6	0.5	
	range	1 – 3	1 – 3	
No. of dental assistants	mean	7.5	6.3	0.08
	SD	3.6	2.9	
	range	1 – 17	1 – 17	

SD, standard deviation.

*Statistical significances of difference: P < 0.05.

### Domains of the European practice assessment

Changes in the scores for the five domains and all dimensions for both the intervention group (first and second assessment) and comparison groups are shown in Table 2. We observed an improvement in quality management in the intervention group, with a change in score of 4.4 encompassing all 281 indicators within the five domains. A higher score occurred in the intervention group in all five domains, particularly in the domains “information” (T0 82.6, T1 89.0) and “quality and safety” (T0 82.4, T1 88.7), which showed an improvement over 6.0. Compared with the comparison group at first assessment, the intervention group at second assessment had significantly better scores for the domains “infrastructure” (T1 94.2, Comparison group 84.0) and “quality and safety” (T1 88.7, Comparison group 78.1).

**Table 2:** Mean scores* for the domains and dimensions of the European practice assessment instrument

**Domain**	**No of indicators**	**Intervention group (n = 45)**		**Change in score (95% CI)**	**Score for comparison group**^**2 **^**(n = 56)**	**Between-group difference in scores**^**3 **^**(95% CI)**
		**Score at first assessment**	**Score at second assessment**			
Infrastructure	77	90.8	94.2	3.4 (−1.9; 8.7)	84.0	10.2 (2.3; 18.1)*
Accessibility and availability	15	76.9	85.5	8.6 (0.4; 16.8)	76.1	9.4 (1.8; 17.0)*
Disabled access	5	55.0	59.2	4.2 (−1.7; 10.1)	57.3	1.9 (−1.7; 5.5)
Premises	20	89.4	92.8	3.4 (−1.9; 8.7)	88.6	4.2 (−1.1; 9.5)
Medical equipment, including drugs	14	89.7	91.7	2.0 (−2.1; 6.1)	83.5	8.2 (1.0; 15.4)*
Nonmedical equipment	3	83.4	90.9	7.5 (−0.2; 15.2)	83.0	7.9 (0.8; 15.0)*
Material management	5	95.9	97.7	1.8 (−2.1; 5.7)	98.9	−1.2 (−4.1; 1.7)
Laboratory management	9	94.7	95.1	0.4 (−1.4; 2.2)	90.4	4.7 (−0.8; 10.2)
IT-security	6	90.8	94.2	3.4 (−1.9; 8.7)	90.5	3.7 (−1.2; 8.6)
People	79	79.9	82.3	2.4 (−2.1; 6.9)	78.1	4.2 (−1.1; 9.5)
Perspective of patients	33	87.3	87.3	---	87.7	−0.4 (−2.1; 1.3)
Perspective of dental staff on working conditions	15	77.5	79.4	1.9 (−2.1; 5.9)	76.7	2.7 (−1.5; 6.9)
Perspective of dentists on working conditions	12	83.0	82.0	−1.0 (−3.9; 1.9)	78.7	3.3 (−1.4; 8.0)
Staff management	10	64.9	77.5	12.6 (2.9; 22.2)	60.2	17.3 (7.4; 27.2)*
Education and training	9	69.7	74.0	4.3 (−1.6; 10.2)	64.7	9.3 (1.7; 16.9)
Information	56	82.6	89.0	6.4 (−0.7; 13.6)	82.0	7.0 (0.3; 13.7)
Confidentiality and privacy	6	85.6	93.6	8.0 (0.01; 15.9)	82.3	11.3 (3.0; 19.6)*
Prevention	8	75.7	79.9	4.2 (−1.7; 10.1)	67.6	12.3 (3.7; 20.9)*
Clinical data, patients records	7	91.6	95 .7	4.1 (−1.7; 9.9)	91.3	4.4 (−0.9; 9.8)
Information for staff	3	81.6	89.5	7.9 (0.01; 15.9)	86.2	3.3 (−1.4; 8.0)
Information for patients on medical care	16	91.9	94.6	2.7 (−2.0; 7.4)	91.4	3.2 (−1.4; 7.8)
Use of computers	2	77.8	87.6	9.8 (1.1; 18.5)	82.9	4.7 (−0.8; 10.2)
Communication with other health care providers	5	79.9	92.7	12.8 (3.3; 22.6)*	84.1	8.6 (1.3; 15.9)*
Information for patients on practice, practice policy and community resources	9	66.3	76.8	10.5 (1.5; 19.5)	67.8	9.0 (1.5; 16.5)*
Finance	10	83.2	86.3	3.1 (−1.9; 8.2)	78.2	8.1 (0.9; 15.2)
Financial leadership and responsibilities	6	90.2	91.3	1.1 (−1.9; 4.1)	87.7	3.6 (−1.3; 8.5)
Financial planning	1	42.2	48.9	6.7 (−0.6; 14.0)	33.3	15.6 (6.1; 25.1)*
Annual report (retrospective)	3	82.9	88.9	6.0 (−0.9; 12.9)	73.5	15.4 (5.9; 24.9)*
Quality & Safety	59	82.4	88.7	6.3 (−0.7; 13.4)	78.1	10.6 (2.5; 18.7)*
Quality development, quality policy	10	76.1	80.5	4.4 (−1.6; 10.4)	69.1	11.4 (3.1; 19.7)*
Detection of quality and safety problems	11	83.1	88.7	5.6 (−1.1; 12.3)	77.4	11.3 (3.0; 19.6)*
Safety of staff and patients, hygiene, infection control	24	93.2	97.0	3.8 (−1.8; 9.4)	91.4	5.6 (−0.4; 11.6)
Provisions for emergency situations	6	74.2	83.3	9.1 (0.7; 17.5)	68.7	14.6 (5.4; 23.8)*
Complaint management	5	54.4	74.4	20.0 (8.3; 31.7)*	48.7	25.7 (14.3; 37.1)*
Analysis of critical incidents	3	78.1	83.3	5.2 (−1.3; 11.7)	72.0	11.3 (3.0; 19.6)*
Total	281	82.7	87.1	4.4 (−1.0; 9.8)	80.5	6.6 (0.1; 13.1)

T0, First assessment; T1, Second assessment.

*Statistical significances of difference: P < 0.05.

^1^Mean scores are on a scale of 0 to 100 and are based on the proportion of indicators for which a positive response was achieved by all of the practices.

^2^Mean scores at first assessment.

^3^Comparison between mean scores at second assessment for intervention practices and mean scores at first assessment for comparative practices since there was no second assessment for this group.

Improvements were observed in the intervention group in the dimensions “complaint management” and “communication with other health care providers”. Compared with the comparison group at first assessment, the intervention group at second assessment had significantly better score in the dimensions such as “medical equipment, including drugs”, “staff management”, “prevention” and “complaint management”.

### Elements associated with each domain of the European practice assessment

Table 3 shows the results of the regression analyses separated for each of the five domains regarding practice characteristics and affiliation to intervention or comparison group. The five regression models explained between 6.4% and 27.2% of the variance respectively. The only independent variable that showed a significant association with each domain was intervention versus comparison group affiliation with the intervention group demonstrating higher scores in each domain.

**Table 3:** Impact of the practice characteristics and group affiliation for each domain (results of linear regression analyses, under specification of standardized beta coefficient and 95% confidence interval (CI), α = 5%)

	**Domains**				
	**Infrastructure**	**People**	**Information**	**Finance**	**Quality & safety**
	β (p-value*) 95% CI	β (p-value*) 95% CI	β (p-value*) 95% CI	β (p-value*) 95% CI	β (p-value*) 95% CI
Mode of practice (0 = single; 1 = group)	−0.050 (0.80) (−6.50; 5.03)	0.034 (0.87) (−4.81; 5:70)	−0.149 (0.45) (−8.70; 3.85)	−0.561 (0.01) (−29.02; −5.37)	−0.014 (0.94) (−7.49; 6.92)
Location of practice (0 = rural; 1 = urban)	−0.016 (0.87) (−2.95; 2.51)	−0.106 (0.31) (−3.78; 1.20)	−0.096 (0.34) (−4.4; 1.53)	0.037 (0.71) (−4.56; 6.64)	−0.112 (0.23) (−5.54; 1.29)
No. of dentists	0.118 (0.57) (−3.51;6.39)	−0.097 (0.64) (−5.56; 3.46)	0.066 (0.75) (−4.51; 6.27)	0.454 (0.03) (1.24; 21.56)	−0.048 (0.80) (−7.00; 5.38)
No. of dental assistants	0.068 (0.56) (−0.33; 0.61)	−0.055 (0.64) (−0.53; 0.33)	0.056 (0.62) (−0.39; 0.64)	0.020 (0.86) (0.55; 1.06)	0.076 (0.47) (−0.37; 0.81)
Group affiliation (0 = intervention group; 1 = comparison group)	−0.379 (<0.01) (−7.97; −2.47)	−0.327 (<0.01) (−6.51; −1.49)	−0.964 (<0.01) (−8.94; −2.95)	−0.295 (0.01) (−14.02; −2.72)	−0.542 (<0.01) (−13.73; −6.85)
Pseudo R^2^	0.116	0.064	0.123	0.121	0.272

*Statistical significance of difference: P < 0.05.

## Discussion

To our knowledge, this is the first study that has evaluated and demonstrated quality improvement in primary dental care practices in Germany. In this study, a repeated measurement was used to evaluate the effect of the assessment process using the *European Practice Assessment* quality management program. The intervention and comparison group practices did not differ remarkably in comparison to general medical practice characteristics. Furthermore, the baseline data and the first assessment of the intervention group showed higher scores than in the comparison group within the five key domains (‘infrastructure’, ‘people’, ‘information’, ‘finance’, and ‘quality and safety’). The comparison of the results of the second assessments in intervention practices with the baseline assessments in comparison group practices showed improvements across all domains, but especially within the domains of ‘quality and safety’ and ‘infrastructure’.

Quality improvement depends on a set of valid and feasible quality indicators that are able to measure quality of care [23]. “Indicators are measurable elements of practice for which there is evidence or consensus that they reflect quality and hence help change the quality of care provided” [23,24]. The implementation of a quality management system in practices can be facilitated by the use of quality indicators. Quality indicators should yield positive assessment on a range of attributes such as clarity, feasibility, reliability, validity and transparency and in order to demonstrate sensitivity to change, benchmarking data are required so that health care providers can assess and compare their own quality of care with others [23,25]. Moreover, for assessment to lead to improvement it must be part of an ongoing process such as the “plan-do-study-act” (PDSA) cycle [26]. A continuous quality improvement is an essential part of quality management programs for health care services, which includes general medical practices and dental care practices in the primary sector [18].

The evaluation of quality of care requires a mixture of objective and subjective measures [27]. The *European Practice Assessment* consists of a set of objective and subjective quality indicators, which evaluate the structure and process of care from the perspective of practice owners, staff, patients and trained external facilitators [17]. For general medical practices, the effectiveness of the *European Practice Assessment* in showing higher scores at repeat assessment has already been shown [18,20]. These results regarding general medical practice are comparable to our results regarding the improvement for each domain at dental care practices [18,20]. The improvement of dimensions and domains in dental care practices follows a similar trend to that of the improvement in general medical practices.

There are different quality improvement activities being initiated in oral health services worldwide ranging from measurements of the process of technical restoration procedures to examination of long term health outcomes for the population [9]. Within this range, one important component is the measurement of dental care practice operations including structure, process and outcomes [9]. However, a systematic and organized agenda for quality improvement in dentistry is still in its fledgling stage [7]. The results of this study suggest that the *European Practice Assessment* provides a much needed mechanism for assessing quality in dental care practices and improving quality and safety [28].

Overall, reliable evidence regarding effectiveness of quality management programs in any field of healthcare is limited [29,30]. Studies on the effectiveness of quality management programs for dental care practices can make an important contribution to the evidence base related to quality in oral health services and also to improving patient outcomes. This is important if oral health services are to stay on par with other health services in terms of quality management [7,31]. Therefore, raising awareness regarding the development and continuous measurement of quality in dental care practices is important for dentists and oral health services policy makers. One opportunity (enabler) would be the introduction of performance-based reimbursement to incentivize good quality of care. The potential role of performance-based reimbursement for dentistry is currently under discussion in the United Kingdom and it is being piloted [3,32]. However, performance-based reimbursement is also associated with unintended consequences [33,34]. For example, the introduction of performance-based reimbursement in general medical care practices has shown short-term gains, but the evidence for its effectiveness long-term is not compelling [35,36]. Therefore, it should be implemented with caution in dental care settings [2,37] and its implementation should only be considered within the context of a system wide quality improvement strategy [9].

### Limitations

Our study has the following limitations. The sample of participating dental care practices was small and may not have been generally representative of dental care practices in Germany. However, all practices that had used the *European Practice Assessment* twice were included. The allocation of practices to an intervention or a comparison group was not randomized and a baseline measurement in the comparison group was lacking. Moreover, the study design has a weakness in that the pre-post measurement was possible with the intervention group, but with only a single set of observations at a second point in time. Although our results showed improvements in the intervention group, this may reflect a selection effect of dental practices volunteering for the first round of the *European Practice Assessment*. Therefore, the results of the study have to be interpreted carefully and need to be confirmed in further studies. In addition, although it is known, that a multifaceted quality management program motivates practices to change [38], there is no reliable evidence from this study about the impact on clinical outcomes because the data presented concentrates on structure and process of care. Because this was the first study evaluating effectiveness of a quality management program in primary dental care settings in Germany, we have no experience on which to base our assessment as to how clinically relevant our results are. At this time, we have no reference standards. This study provides preliminary results as basis for further studies. The study design was explorative. Therefore, no correction for multiple tests was needed. The observed effects should be examined in further study with a larger sample.

## Conclusions

In summary, implementation of quality management in dental care practices requires a paradigm shift: there cannot longer be a singular focus on technical aspects, but also it is necessary to integrate organizational aspects of service delivery and employ a team approach. Based on results from this study, this has the potential to result in better organizational quality in dental care practices. The *European Practice Assessment* for dental care practices provides such a quality management program, as it focuses on the improvement of structural and organizational aspects to promote high quality of care.

## Competing interests

BB and MB are employed by the AQUA-Institute which disseminates the *European Practice Assessment* in Germany. JS is its director and stockholder. Other authors: No conflict of interest declared.

## Authors’ contributions

KG, SC and JS initiated and designed the study. MB and BB coordinated the study. KG carried out data analysis and wrote the manuscript. All authors (KG, SC, MB, BB, MW and JS) read earlier versions of the manuscript, provided critical comments and approved the final manuscript.

## Pre-publication history

The pre-publication history for this paper can be accessed here:

http://www.biomedcentral.com/1472-6831/14/41/prepub

## References

[B1] World Health Organization (WHO) Quality and accreditation in health services. A global review 2003 Geneva (Switzerland): The Organization

[B2] The Institute of Medicine (IOH) Crossing the quality chasm: a new health system for the 21st century 2001 Washington D.C: National Academies Press 11549951

[B3] Mills I Batchelor P Quality indicators: the rationale behind their use in NHS dentistry Br Dent J 2011 211 11 15 10.1038/sj.bdj.2011.521 21738180

[B4] Donabedian A The quality of care. How can it be assessed? JAMA 1988 260 1743 1748 10.1001/jama.1988.03410120089033 3045356

[B5] Campbell SM Roland MO Buetow S Defining quality of care Soc Sci Med 2000 51 1611 1625 10.1016/S0277-9536(00)00057-5 11072882

[B6] Campbell SM Tickle M What is quality primary dental care? Br Dent J 2013 215 135 139 10.1038/sj.bdj.2013.740 23928610

[B7] Bader JD Challenges in quality assessment of dental care J Am Dent Assoc 2009 140 1456 1464 10.14219/jada.archive.2009.0084 19955057

[B8] Greenfield D Braithwaite J Health sector accreditation research: a systematic review Int J Qual Health Care 2008 20 172 183 10.1093/intqhc/mzn005 18339666

[B9] Glassman P Oral health quality improvement in the era of accountability 2011 Michigan: W.K. Kellogg Foundation

[B10] Kenny DJ Conway RM Johnston DH The development of IS09002 quality management standards for Canadian dental practices J Can Dent Assoc 1999 65 105 108 10079620

[B11] NHS Scotland An Action Plan for Improving Oral Health and Modernising NHS Dental Services in Scotland 2005 Scottish Executive Edinburgh http://www.scotland.gov.uk/Resource/Doc/37428/0012526.pdf. [last accessed April 24, 2014]

[B12] Crall JJ Spritzer KL Hays RD Development and implementation of a dental office assessment program J Am Coll Dent 2012 79 33 41 23189803

[B13] Deming EW Massachusetts Institute of Technology, Centre for Advanced Engineering Study 1986 Cambridge (MA): Out of Crisis

[B14] Federal Joint Committee (GB-A) Quality management guideline for dental care 2006 http://www.eazf.de/doc/Dokumente/QM-Richtlinie_GBA.pdf. [last accessed April 24, 2014]

[B15] Bergmann Krauss B Boehme P Qualitätsmanagement-Systeme für die Zahnarztpraxis IDZ-Inform 2005 5 4 38 in German

[B16] Mettes TG van der Sanden WJ Bronkhorst E Grol RP Wensing M Plasschaert AJ Impact of guideline implementation on patient care: randomized trial J Dent Res 2009 89 71 76 19966044 10.1177/0022034509350971

[B17] Engels Y Dautzenberg M Campbell S Broge B Boffin N Marshall M Elwyn G Vodopivec-Jamsek V Gerlach FM Samuelson M Grol R Testing a European set of indicators for the evaluation of the management of primary care practices Fam Pract 2006 23 137 147 16243953 10.1093/fampra/cmi091

[B18] Szecsenyi J Campbell S Broge B Laux G Willms S Wensing M Goetz K Effectiveness of a quality-improvement program in improving management of primary care practices CMAJ 2011 183 E1326 E1333 10.1503/cmaj.110412 22043000 PMC3255110

[B19] EPA Website European Practice Assessmenthttp://www.epa-qm.de/epa/front_content.php?idart=218. [last accessed April 24, 2014]

[B20] Götz K Szecsenyi J Broge B Willms S [Welche Wirkung hat Qualitätsmanagement in Arztpraxen? Ergebnisse aus Entwicklung und Evaluation des Europäischen Praxisassessments (EPA).] 2011 Göttingen: AQUA-Verlag in German

[B21] AQUA-Website Institute for Applied Quality Improvement and Research in Health Carehttp://www.aqua-institut.de. [last accessed April 24, 2014]

[B22] Goderis G Borgermans L Heyrman J Van Den Broeke C Carbonez A Mathieu C Verbeke G Grol R Monitoring modifiable cardiovascular risk in type 2 diabetes care in general practice. The use of an aggregated z-score Med Care 2010 48 589 595 10.1097/MLR.0b013e3181d5693a 20562687

[B23] Campbell SM Braspenning J Hutchinson A Marshall M Research methods used in developing and applying quality indicators in primary care Br Med J 2003 326 816 819 10.1136/bmj.326.7393.816 12689983 PMC1125721

[B24] Lawrence M Olesen F Indicators of quality health care Eur J Gen Pract 1997 3 103 108 10.3109/13814789709160336

[B25] Braspenning J Hermans R Calsbeek H Westert G Campbell SM Grol R Grol R, Wensing M, Eccles M, Davis D Quality and safety of care: the role of indicators Improving patient care: the implementation of change in health care practice 2013 2 Oxford: UK John Wiley & Sons, Ltd

[B26] Boaden R Harvey G Moxham C Proudlove N Quality improvement: theory and practice in healthcare 2008 Coventry: NHS Institute for Innovation and Improvement

[B27] Campbell SM Eriksson T Multiple strategies for quality improvement and patient safety – money alone is not the answer, nor is trust. Conclusions of the 6th April 2011 Euro J Gen Pract 2011 17 238 240 10.3109/13814788.2011.602669 22111552

[B28] Lester HE Eriksson T Dijkstra R Martinson K Tomasik T Sparrow N Practice accreditation: the European perspective Br J Gen Pract 2012 62 273 274 10.3399/bjgp12X641627 PMC3338063 22546601

[B29] Minkman M Ahaus K Huijsman R Performance improvement based on integrated quality management models: what evidence do we have? A systematic literature review Int J Qual Health Care 2007 19 90 104 10.1093/intqhc/mzl071 17277010

[B30] Schouten LMT Hulscher MEJL Everdingen JJE V Huijsman R Grol R Evidence for the impact of quality improvement collaboratives: systematic review BMJ 2008 336 1491 1494 10.1136/bmj.39570.749884.BE 18577559 PMC2440907

[B31] Niederman R Leitch J “Know what” and “Know how”: knowledge creation in clinical practice J Dent Res 2006 85 296 297 10.1177/154405910608500403 16567547

[B32] Voinea-Griffin A Fellows JL Rindal DB Barasch A Gilbert GH Safford MM Pay for performance: will dentistry follow? BMC Oral Health 2010 10 9 10.1186/1472-6831-10-9 20423526 PMC2880362

[B33] Campbell SM Kontopantelis E Hannon KL Burke M Barber A Lester HE Framework and indicator testing protocol for developing and piloting quality indicators for the UK Quality and Outcomes Framework BMC Fam Pract 2011 12 85 10.1186/1471-2296-12-85 21831317 PMC3176158

[B34] Tickle M Campbell SM How do we measure quality in primary dental care? Br Dent J 2013 215 183 187 10.1038/sj.bdj.2013.789 23969661

[B35] Petersen LA Woodard LD Urech T Daw C Sookanan S Does Pay-for-Performance improve the quality of health care? Ann Intern Med 2006 145 265 272 10.7326/0003-4819-145-4-200608150-00006 16908917

[B36] Scott A Sivey P Ait Ouakrim D Willenberg L Naccarella L Furler J Young D The effect of financial incentives on the quality of health care provided by primary care physicians Cochrane Database Syst Rev 2011 9 CD008451 21901722 10.1002/14651858.CD008451.pub2

[B37] Campbell SM Tickle M How do we improve quality in primary dental care? Br Dent J 2013 215 239 243 10.1038/sj.bdj.2013.831 24029991

[B38] Grimshaw J McAuley LM Bero LA Grilli R Oxman A Ramsay C Vale L Zwarenstein M Systematic reviews of the effectiveness of quality improvement strategies and programmes Qual Saf Health Care 2003 12 298 303 10.1136/qhc.12.4.298 12897365 PMC1743751

